# IDDNet: Infrared Object Detection Network Based on Multi-Scale Fusion Dehazing

**DOI:** 10.3390/s25072169

**Published:** 2025-03-29

**Authors:** Shizun Sun, Shuo Han, Junwei Xu, Jie Zhao, Ziyu Xu, Lingjie Li, Zhaoming Han, Bo Mo

**Affiliations:** 1 School of Aerospace Engineering, Beijing Institute of Technology, Beijing 100081, China; 3220225009@bit.edu.cn (S.S.); 3220215010@bit.edu.cn (S.H.); 3120220104@bit.edu.cn (J.X.); zhaojie2103@163.com (J.Z.); 3120230077@bit.edu.cn (Z.X.); 17870087133@163.com (L.L.); 2 North Navigation Control Technology Co., Ltd., Beijing 101102, China; 3 School of Computer Science & Technology, Henan Institute of Technology, Xinxiang 453003, China; zhaoming_han95@163.com

**Keywords:** dehazing, infrared object detection, feature fusion, attention mechanism, deep learning

## Abstract

In foggy environments, infrared images suffer from reduced contrast, degraded details, and blurred objects, which impair detection accuracy and real-time performance. To tackle these issues, we propose IDDNet, a lightweight infrared object detection network that integrates multi-scale fusion dehazing. IDDNet includes a multi-scale fusion dehazing (MSFD) module, which uses multi-scale feature fusion to eliminate haze interference while preserving key object details. A dedicated dehazing loss function, DhLoss, further improves the dehazing effect. In addition to MSFD, IDDNet incorporates three main components: (1) bidirectional polarized self-attention, (2) a weighted bidirectional feature pyramid network, and (3) multi-scale object detection layers. This architecture ensures high detection accuracy and computational efficiency. A two-stage training strategy optimizes the model’s performance, enhancing its accuracy and robustness in foggy environments. Extensive experiments on public datasets demonstrate that IDDNet achieves 89.4% precision and 83.9% AP, showing its superior accuracy, processing speed, generalization, and robust detection performance.

## 1. Introduction

In infrared image-guided tasks, the accuracy of infrared object detection is a critical factor determining the success of the guidance mission [1]. In battlefield environments, unmanned aerial vehicles (UAVs) often operate in complex and dynamic scenes, frequently encountering interference from haze, smoke, and dust. These factors significantly degrade the contrast of infrared images, leading to substantial loss of detail information, which in turn affects the accuracy and robustness of object detection algorithms [2,3,4] in complex backgrounds. Consequently, object detection models for hazy environments have garnered significant research interest and attention.

However, existing approaches [5,6,7,8,9,10,11,12,13,14,15,16,17,18,19,20,21,22,23,24,25] still exhibit notable limitations in practical applications. While physics-based methods demonstrate reasonable generalization through manually designed priors like dark channel and haze line assumptions [6,9,13], their reliance on specific statistical hypotheses often leads to performance degradation in complex real-world scenarios. The inherent characteristics of infrared images, such as low contrast, lack of color information, and limited dynamic range [17], make these challenges even more difficult. Moreover, deep learning-based alternatives, though data-driven, require extensive training datasets and frequently fail to adequately address the unique degradation patterns of infrared sensors in hazy conditions [18,19,20,21,22,23,24,25].

In single-image infrared dehazing tasks, early solutions, such as dark channel prior (DCP) [6], color attenuation prior [8], non-local prior [9], contrast maximization [12], and haze line prior [13], estimate the transmission map by manually analyzing image data, then decompose the image into reflection and transmission components to remove haze-induced blurring. For example, He et al. [14] introduced guided filtering to replace soft matting, while Li et al. [15] improved quadtree subdivision to reduce estimation errors in local highlights. Dong et al. [16] proposed a dehazing method based on grayscale variance and average gradient, effective for hazy and underwater environments. Infrared images in hazy conditions often exhibit low contrast and dynamic range, leading Galdran [17] to use multi-exposure image fusion techniques to adjust contrast and saturation for obtaining a clearer image.

Deep learning-based dehazing methods overcome the limitations of traditional prior-based approaches. Tang et al. [7] trained a random forest model on synthetic datasets, while CNN-based methods [11,18,19,20], such as DehazeNet [10] and GAN-based models [21], advanced dehazing through end-to-end feature learning. Zhu et al. [22] proposed a fast dehazing algorithm using multi-exposure image fusion, and Ma et al. [23] introduced the temporal information injection network (TIIN), which enhances haze removal by leveraging temporal information. However, many CNN-based methods still require separate estimation of atmospheric light or transmission maps. To address this, Zhang et al. [20] introduced a dense connected pyramid dehazing network (DCPDN), using pyramid pooling for multi-scale feature learning, and Li et al. [24] proposed a conditional GAN (cGAN) for end-to-end dehazing. Despite their strong performance, cGAN and DCPDN suffer from large model sizes (>200 MB), making them impractical for real-time dehazing on resource-constrained platforms like aerial vehicles. Liu et al. [25] achieved flexible dehazing for high-resolution visible light images using a multi-scale densely connected network, but its performance on infrared images has certain limitations.

Most existing methods focus on the integration of visible light dehazing and detection, while studies on infrared dehazing and detection remain limited. Current research often treats infrared dehazing and detection separately or simply stacks them together, overlooking the potential synergy between the two [26,27]. For instance, Wang et al. [28] proposed MDD-ShipNet for ship detection using a CNN-based dehazing mechanism, while Liu et al. [29] developed IA-YOLO to enhance images for optimized detection. Meng et al. [30] introduced YOLOv5s-Fog, improving feature extraction with SwinFocus and replacing traditional NMS with Soft-NMS.

In summary, methods based on physical priors use prior knowledge and physical models to restore image clarity but struggle in complex or dynamic scenes. Deep learning-based methods have advanced in dehazing and detection, yet their complexity and reliance on large datasets limit their real-world applicability, especially in infrared images with uneven fog distribution. Furthermore, current infrared image dehazing methods, largely based on techniques from visible light dehazing, struggle with uneven fog distribution, often leading to incomplete dehazing and inaccurate detections.

This paper proposes IDDNet, a fast, accurate, and lightweight network for single-image infrared dehazing and detection. It integrates a multi-scale fusion dehazing module with a multi-scale object detection network, enabling direct dehazing and detection of foggy images while optimizing module collaboration and ensuring real-time performance. Using lightweight DSConv convolutions, IDDNet achieves high efficiency with a compact model size. The main contributions are summarized as follows:

1. A multi-scale fusion dehazing (MSFD) module is proposed, based on the atmospheric scattering model. It alternately processes features from different levels through multi-scale feature fusion, effectively removing haze interference while preserving the details and features of the objects.
2. Based on MSFD, an infrared dehazing object detection network, IDDNet, is constructed. IDDNet integrates a bidirectional polarized self-attention mechanism, a weighted bidirectional feature pyramid network, and a multi-scale object detection layer.
3. To address the issues of texture loss and high noise in infrared dehazing, this paper designs a dehazing loss function (DhLoss) that integrates joint perceptual loss, contrast loss, and smoothness loss. Additionally, a dehazing object detection loss function (DetLoss) is constructed by incorporating scale loss and location loss to enhance detection accuracy and robustness in complex backgrounds.

Extensive experiments on public datasets, including infrared dehazing verification, infrared object detection validation, and generalization experiments, show that the proposed network outperforms state-of-the-art methods in dehazing effectiveness and detection accuracy while demonstrating stability and reliability.

## 2. Related Works

### 2.1. Atmospheric Scattering Model

**Figure 1:**
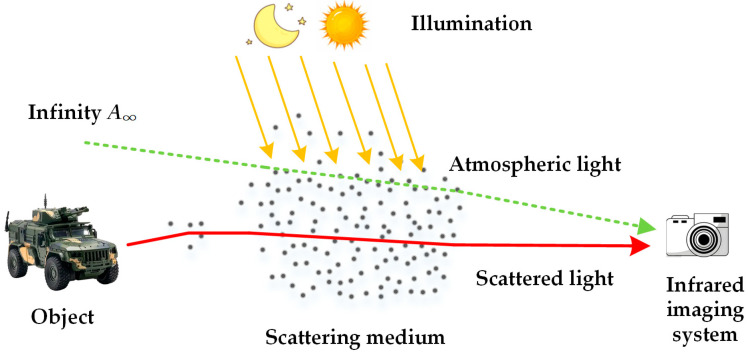
The light sources received by the infrared imaging system under foggy conditions.

The atmospheric scattering model (ASM) [31,32] explains light interaction with haze, supporting the degradation mechanism of foggy images. As shown in Figure 1, infrared imaging receives light from two sources: scattered object reflections attenuated by the air and atmospheric light formed by ambient and illumination scattering.

The expression of foggy imaging obtained through ASM is shown in Equation (1),

(1) I(x,λ)=J(x,λ)⋅t(x,λ)+A(x,λ)(1−t(x,λ)),

where *x* represents the position of a pixel point, and λ represents the wavelength of infrared light; I(x,λ) represents the infrared foggy image obtained by the imaging system; J(x,λ) is the restored infrared fog-free image; t(x,λ) denotes the transmission function, which represents the proportion of light that reaches the infrared imaging system after attenuation; and A(x,λ) is the atmospheric light component in the infrared image.

Most restoration studies use ASM (Equation (1)) to describe image degradation. Some estimate the transmission map with manually designed priors, while others use learning methods to predict both the transmission map and the clear image from foggy ones. In recent years, reconstructing Equation (1) and rapidly estimating dehazing parameters have become key areas for improving dehazing performance. For example, Li et al. [18] introduced an estimator K to solve for the clear image and proposed an end-to-end lightweight atmospheric optical depth network (AOD-Net) based on an improved ASM. Similar designs are found in [33,34], where [33] used a multi-scale pyramid architecture, and [34] proposed a feature-level dehazing module. Ju et al. [35] introduced scene reflectance to help the model produce results with appropriate illumination and minimal color shifts.

### 2.2. Detection Model

**Figure 2:**
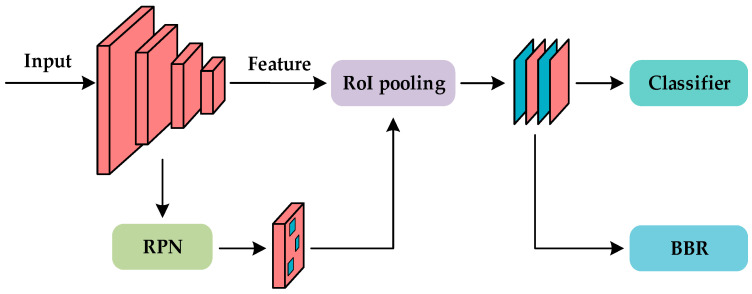
The structure of Faster R-CNN. RoI stands for region of interest; RPN stands for region proposal network; BBR stands for bounding box regression.

Deep learning-based object detection models are categorized as single-stage (e.g., YOLO [36,37,38], RetinaNet [39]) or two-stage (e.g., Faster R-CNN, Mask R-CNN, CascadeR-CNN [40,41,42]) based on the use of region proposals. R-CNN [43] pioneered deep learning for object detection, while Faster R-CNN [40] improved efficiency by introducing the region proposal network (RPN) for automatic candidate region generation. As shown in Figure 2, Faster R-CNN generally outperformed earlier single-stage models in terms of detection accuracy.

With advancements in single-stage detection (e.g., YOLO, RetinaNet), performance rivaled or surpassed two-stage methods while maintaining real-time efficiency. RetinaNet, built on ResNet and the feature pyramid network (FPN), used Focal Loss to adjust sample weights, while YOLO improved detection and speed through iterative updates.

## 3. Materials and Methods

### 3.1. Multi-Scale Fusion Dehazing Module

The ASM is introduced in the related work. In Equation (1), the transmittance t(x,λ) is as shown in Equation (2),

(2) $$t(x,\lambda )=e^{-\beta (\lambda )d(x)},$$

where *β* is the atmospheric scattering coefficient, representing the density of the fog; *d*(*x*) is the depth of field, representing the distance from the infrared imaging system to the scene point. It can be seen from Equation (2) that when *d*(*x*) approaches infinity, t(x,λ) approaches zero, resulting in blurred actual imaging and loss of detailed textures. Combining with Equation (1), we can obtain the following:

(3) $$\underset{d(x,\lambda )\to \infty }{\lim}t(x,\lambda )=0,\;A(x,\lambda )=I(x,\lambda ).$$

The inference of the above imaging process reveals the reasons for the degradation of foggy infrared images. By performing inverse inference on Equation (1), a clear, fog-free image J(x,λ) can be obtained, and its expression is shown in Equation (4).

(4) $$J(x,\lambda )=\frac{1}{t(x,\lambda )}I(x,\lambda )-\frac{1}{t(x,\lambda )}A(x,\lambda )+A(x,\lambda ).$$

The separate estimation of the transmission t(x,λ) and atmospheric light A(x,λ) often leads to significant errors due to the complex coupling relationship between variables and the non-uniform distribution of the environment. Based on AOD [18], this paper introduces a bias term *b* to reduce the impact of potential errors. These two parameters are modeled as a scaling factor ω, defined as shown in Equation (5).

(5) $$\omega =\frac{1}{t(x,\lambda )}\left(\frac{I(x,\lambda )-A(x,\lambda )}{I(x,\lambda )-1}\right)+\frac{\left(A(x,\lambda )-b\right)}{I(x,\lambda )-1}.$$

The final infrared dehazed image J(x,λ) is obtained as shown in Equation (6),

(6) J(x,λ)=ωI(x,λ)−ω+b,

where the scaling factor ω is used to correct the light attenuation effect caused by haze, minimizing the impact of image loss. The bias term b serves as a global compensation parameter to correct pixel deviations caused by uneven ambient light distribution or local light intensity anomalies.

**Figure 3:**
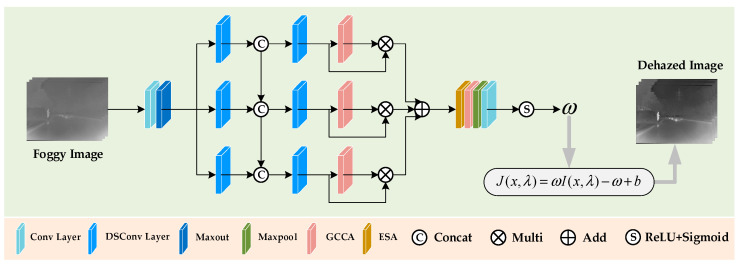
The structure of MSFD; the pink-highlighted areas display the components of MSFD.

This paper proposes a lightweight multi-scale fusion dehazing (MSFD) module, as shown in Figure 3. Unlike traditional methods that rely on complex calculations, MSFD directly estimates the scaling factor for infrared images, avoiding separate transmission and atmospheric light computations. It efficiently dehazes images while preserving key details, structure, edges, and textures.

**Figure 4:**
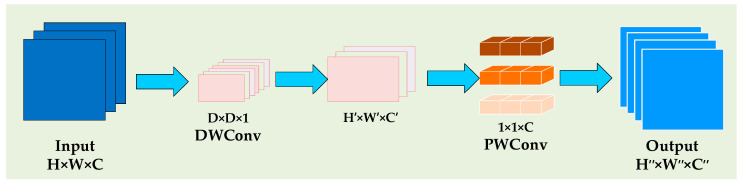
The structure of DSConv.

The Maxout operation reduces feature dimensionality and performs group fusion. After a 1 × 1 convolution, Maxout applies group-based linear transformations to multiple input channels, generating task-adaptive feature representations. This operation minimizes redundancy and prepares input features for the multi-scale DSConv module. DSConv, the core of MSFD, combines depthwise convolution (DWConv) to extract spatial features and pointwise convolution (PWConv) for channel features. Compared to standard convolution, DSConv reduces parameter count and computational complexity, enhancing efficiency. The structure of DSConv is shown in Figure 4.

Assuming the input feature map size is *H* × *W* × *C*, the depthwise convolution kernel size is *D* × *D* × 1 with *C* channels, and the pointwise convolution kernel size is 1 × 1 × *C* with *K* kernels, the ratio ϕ of the number of parameters in DSConv convolution to the number of parameters in standard convolution is as follows:

(7) $$\phi =\frac{P_{\mathrm{DSConv}}}{P_{\mathrm{Conv}}}=\frac{D\times D\times 1\times C+1\times 1\times C\times K}{D\times D\times C\times K}=\frac{1}{K}+\frac{1}{D^{2}}.$$

The ratio φ of the computational complexity is as follows:

(8) $$\varphi =\frac{F_{\mathrm{DSConv}}}{F_{\mathrm{Conv}}}=\frac{H\times W\times D\times D\times 1\times C+1\times 1\times C\times K\times H\times W}{H\times W\times D\times D\times C\times K}=\frac{1}{K}+\frac{1}{D^{2}}.$$

This paper constructs the MSFD module using DSConv convolutions of sizes 3 × 3, 5 × 5, and 7 × 7, aiming to reduce memory resources required for feature computation and improve training efficiency. The feature extraction principle is shown in Equation (9),

(9) $$F_{k\times k}(I)=DSConv_{k\times k}(I),\;k\in \{3,5,7\},$$

where $DSConv_{k\times k}$ represents the DSConv convolution of k×k, and $F_{k\times k}(I)$ represents the feature map at the corresponding scale.

The dehazing process of MSFD involves three main stages: feature extraction, multi-scale feature fusion, and parametric restoration. First, the network extracts global features from the foggy image using 1 × 1 convolution and Maxout, reducing redundancy and computational complexity. Next, DSConv with varying kernel sizes (3 × 3, 5 × 5, and 7 × 7) captures multi-scale information, and inter-layer cross-fusion integrates these features for comprehensive representation. Four contrast-aware channel attention [44,45] (CCA) modules, enhanced by GSConv and named GCCA, focus on key regions during multi-scale fusion to improve dehazing. The first three GCCAs optimize gradient flow via skip connections, while the fourth, combined with the ESA [46] module, aggregates multi-level feature information. In the final stage, max pooling, convolution, and non-linear activations (ReLU and Sigmoid) compute dehazing parameters to restore a clear infrared image.

#### 3.1.1. GCCA Block

**Figure 5:**
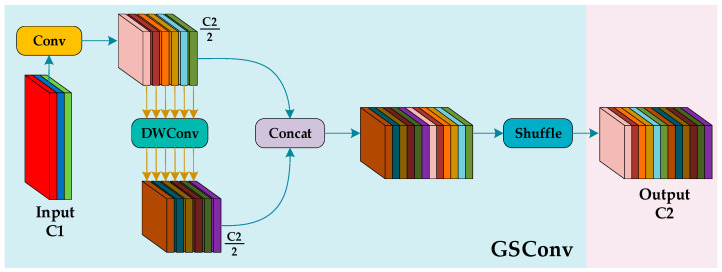
The structure of GSConv. Shuffle refers to randomly rearranging the feature map.

Aircraft systems have limited storage and computational resources, making lightweight network architectures essential for infrared dehazing object detection. Introducing lightweight modules [47,48] to replace large components helps create a more compact model. In this study, a lightweight convolutional structure, GSConv (Figure 5) [48], is introduced to enhance the CCA block, reducing model size while maintaining dehazing performance. This improved module is termed GCCA. To prevent deeper networks from impeding spatial information flow and affecting inference speed, the GCCA block is placed after feature extraction.

**Figure 6:**
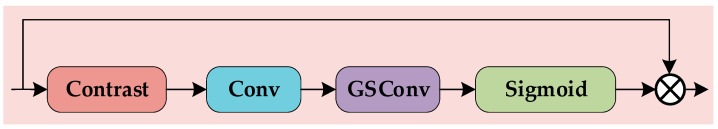
The structure of the GCCA block. GCCA extracts contrast information from the input feature map through the contrast module to enhance feature saliency. Then, standard convolution and GSConv are used to extract high-level semantic features and compress redundant information.

The CCA block is an efficient attention mechanism that effectively addresses the uneven fog distribution in infrared foggy images, enhancing focus on key regions and improving dehazing performance. By incorporating GSConv, the CCA structure is made more lightweight, and the improved GCCA block structure is shown in Figure 6.

The GCCA block uses the Sigmoid activation function to normalize the convolution result; an element-wise multiplication operation is applied to multiply the weights with the input feature map, highlighting the feature representation of key regions and suppressing irrelevant information. The formula for the GCCA block is shown as Equation (10),

(10) $$F_{k\times k}^{GCCA}=\sigma (GSConv(Conv(Contrast(F_{k\times k}(I)))))⊙F_{k\times k}(I),\;k\in \{3,5,7\},$$

where $F_{k\times k}(I)$ represents the input feature map; Contrast(·) represents the contrast information extraction operation; σ(·) represents the Sigmoid activation function; ⊙ represents the element-wise multiplication operation, and $F_{k\times k}^{GCCA}$ represents the output feature map. GCCA adaptively adjusts the weights of each channel to enhance the network’s focus on key features and improve multi-scale feature fusion.

#### 3.1.2. Loss Function of MSFD

To achieve good dehazing performance with MSFD, this paper designs a dedicated dehazing loss function, DhLoss, consisting of perceptual loss, contrast loss, and smoothness loss. The perceptual loss (*L_p_*) utilizes high-level features extracted by VGG16 to enforce semantic structural consistency between dehazed and hazy inputs, preserving critical texture details. The contrast loss (*L_c_*) minimizes local contrast variance to amplify foreground-background differentiation, thereby enhancing global contrast and target discernibility. The smoothness loss (*L_g_*) applies gradient constraints to suppress edge artifacts while maintaining detail continuity and natural transitions. These components are integrated into a composite DhLoss function, achieving coordinated optimization of detail fidelity, target enhancement, and visual smoothness for reliable dehazing performance.

The perceptual loss $L_{p}$ is used to measure the semantic consistency between the dehazed image J(x,λ) and the input foggy image I(x,λ). Its definition is shown in Equation (11),

(11) $$L_{p}=\frac{1}{n}{\sum_{i}\frac{1}{H_{i}W_{i}C_{i}}\left\|\psi_{i}(J(x,\lambda ))-\psi_{i}(I(x,\lambda ))\right\|}_{2}^{2},$$

where $\psi_{i}(\cdot )$ represents the feature map extracted from the *i*-th layer of the pre-trained network, with feature map dimensions of $H_{i}\times W_{i}\times C_{i}$; and *n* represents the number of extracted feature maps.

To further improve the dehazing performance of infrared images, this paper introduces contrast loss $L_{c}$, which utilizes the contrast characteristics of infrared images to highlight the object regions. The definition of the contrast loss is shown in Equation (12),

(12) $$L_{c}=-Var(J(x,\lambda )),$$

where Var(·) represents the local contrast variance of the image pixels.

To improve the continuity and smoothness of the image gradients, this paper introduces a smoothness loss $L_{g}$. By constraining the gradient variations in the horizontal and vertical directions, it achieves refined edge processing and detail restoration in the dehazed image. The definition of the smoothness loss $L_{g}$ is shown in Equation (13),

(13) $$L_{g}={\left\|\nabla_{x}J(x,\lambda )\right\|}_{1}+{\left\|\nabla_{y}J(x,\lambda )\right\|}_{1},$$

where $\nabla_{x}$ and $\nabla_{y}$ represent the gradient variations in the image in the horizontal and vertical directions used to characterize the edge smoothness and detail continuity of the image.

Finally, $L_{p}$, $L_{c}$, and $L_{g}$ together form the loss function DhLoss of the dehazing model, as shown in Equation (14),

(14) $$L_{DhLoss}=\lambda_{1}L_{p}+\lambda_{2}L_{c}+\lambda_{3}L_{g},$$

where $\lambda_{1}$, $\lambda_{2}$, and $\lambda_{3}$ are the weight coefficients of the respective loss terms, adjusted through experiments to suit the infrared dehazing object detection task. After extensive experiments, these are set to $\lambda_{1}=1$, $\lambda_{2}=0.2$, and $\lambda_{3}=0.4$.

Researchers should explore the values of $\lambda_{1}$, $\lambda_{2}$, and $\lambda_{3}$ based on the specific requirements of the dataset and experimental scenario. We recommend $\lambda_{1},\lambda_{2},\lambda_{3}\in (0,1)$ and conducting parameter exploration experiments with a step size of 0.1.

#### 3.1.3. ESA Block

**Figure 7:**
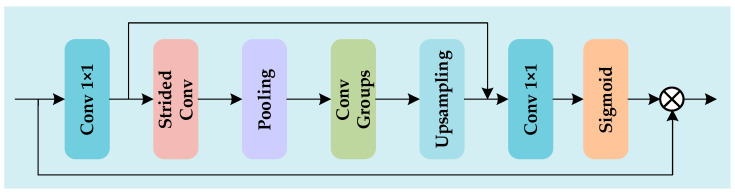
The structure of the ESA block.

To address the contrast degradation caused by haze in infrared images, the ESA block achieves efficient dehazing through lightweight structural design and large receptive field mechanisms. By dynamically focusing on haze-occluded critical regions (e.g., edges, textures) via attention mechanisms, this module enhances local feature representation while constructing global haze distribution models. Integrated with multi-scale feature aggregation strategies, it effectively restores high-frequency details while maintaining real-time processing speed, significantly improving scene clarity and physical authenticity in dehazed images. The structure of the ESA block is shown in Figure 7.

ESA is designed with lightweight principles to minimize computational overhead while ensuring efficient network operation. It expands the receptive field by combining stride convolution with large-window max pooling, capturing broader contextual information and addressing long-range dependency modeling. A 1 × 1 convolution reduces channel dimensions at the input, lowering computational cost while maintaining focus on key features. Stride convolution and max pooling further reduce spatial dimensions, emphasizing important image regions. Finally, upsampling and Sigmoid activation generate attention masks, enhancing the representation of key regions.

### 3.2. Infrared Dehazing Object Detection Network

**Figure 8:**
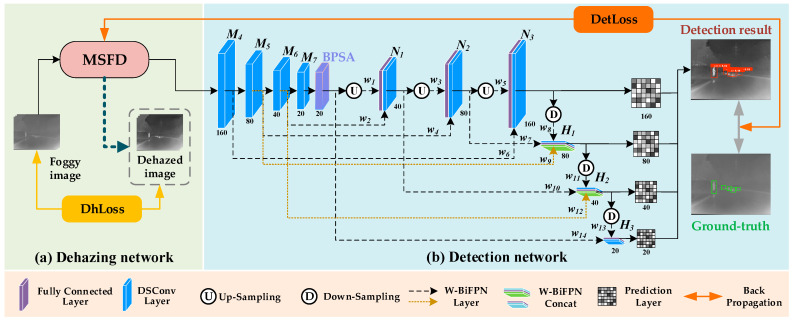
The structure of IDDNet, with the pink-highlighted areas, displays its components, which include a dehazing stage and an object detection stage. The numbers 20, 40, 80, and 160 represent the different feature map sizes at various stages of the algorithm’s processing.

This paper proposes IDDNet, an innovative infrared dehazing object detection model designed for foggy scenes. IDDNet uses MSFD as the dehazing module and integrates the bidirectional polarized self-attention (BPSA), weighted bidirectional feature pyramid network (W-BiFPN), and multi-scale object detection layers, built in a weakly supervised manner. The dehazing loss function (DhLoss) ensures efficient dehazing, while the object detection loss function (DetLoss) guarantees high-quality detection in foggy environments. The structure of IDDNet, shown in Figure 8, demonstrates how joint training of the dehazing and detection modules enhances performance in foggy conditions.

The dehazing stage in MSFD extracts multi-scale features using depthwise separable convolutions, followed by fusion through operations like Concat, Multi, and Add. GCCA and ESA blocks optimize feature weights and enhance focus on key regions. In object detection, the dehazed image is processed using DSConv, with the W-BiFPN structure efficiently fusing multi-scale features. Upsampling and downsampling generate multi-scale feature maps (*N*_1_, *N*_2_, *N*_3_, *H*_1_, *H*_2_, and *H*_3_), while BPSA improves global semantic representation and object localization. These multi-scale maps are input into detection layers for accurate object detection at various scales. The training process integrates DhLoss and DetLoss, utilizing a two-stage strategy to boost both dehazing and detection performance.

The training process of IDDNet is shown in Algorithm 1.
**Algorithm 1.** IDDNet Pseudo-Code**Input:** Haze set (**I_haze_**), Ground truth set (**I_GT_**), Dehaze images (**I_dehaze_**), initial parameters of Dehaze Model Θ_dehaze_, initial parameters of Detection Model Θ_detection_, Learning rate (*η*), Batch size (*B*), Epochs (*N*_epochs_), Loss weights (*L*_DhLoss_, *L*_DetLoss_).**Output:** Optimized model.1: **for** *n* = 1 **to** *N*_epochs_ **do** 2:   **for** *m* = 1 **to** *B* **do** 3:   //**Stage1**. Multi-scale Fusion Dehaze Model (**MSFD**) 4:   Feature extraction: $F_{k\times k}(I)$ ← Maxout and DSConv 5:   Feature fusion: $F_{k\times k}^{GCCA}$ ← Concat, Multi, Add, ESA and GCCA 6:   Dehazing factor: *ω ←* ReLU, Sigmoid and *ω* 7:   *L*_DhLoss_ ← DhLoss (**I_haze_**_,_
**I_dehaze_**) 8:   Θ_dehaze_ ← Θ_dehaze_ ↔ η*∇ ($L_{p}$ + $L_{c}$ + $L_{g}$) 9:   //**Stage2.** Infrared Dehazing Detection Network (**IDDNet**) 10:   BPSA ← (Channel-direction PA + Spatial-direction PA): 11:           $C_{PA}=S_{g}[W_{3}(S_{m}(\delta_{2}(G_{r}))⊗\delta_{1}(G_{t}))]⊙X_{P}$ 12:           $S_{PA}=S_{g}[\delta_{3}(\delta_{2}(G_{w})⊗S_{m}(\delta_{1}(F_{GP}(G_{h}))))]⊙X_{P}$ 13:   BPSA Fusion: $Y_{BPSA}=C_{PA}⊕S_{PA}$ 14:   W-BiFPN feature fusion module: *N*_1,_
*N*_2,_
*N*_3,_
*H*_1,_
*H*_2_ and *H*_3_ 15:   Detection Head ← (*N*_3_, *H*_1_, *H*_2_ and *H*_3_) 16:   *L*_DetLoss_ ← (**I_dehaze_**, **I_GT)_**) 17:   Θ_detection_ ← Θ_detection_ ↔ η*∇ ($L_{sca}$ + $L_{loc}$) 18:   end 19: end

#### 3.2.1. Weighted Bidirectional Feature Pyramid Network

To address the detail dilution issue in IDDNet feature extraction (e.g., edge/texture information loss leading to degraded detection performance) for images processed by MSFD algorithms, this paper proposes a weighted bidirectional feature pyramid network (W-BiFPN) designed for aerial infrared object detection scenarios. The module enhances the bidirectional feature pyramid network [49] through three key innovations: cross-scale bidirectional connections and adaptive weight allocation mechanisms strengthen attention focusing on critical target regions (e.g., aircraft contours, thermal signatures) in multi-scale features, mitigating feature blurring caused by residual haze; DSConv is introduced to reduce computational complexity while preserving feature representation capability; the network hierarchy and dynamic feature fusion mechanism are optimized, improving weight allocation strategies to enhance detection robustness for low-contrast objects in complex hazy environments.

Specifically, the output features of MSFD are passed to the detection network, where four convolutional layers, *M*_4_, *M*_5_, *M*_6_, and *M*_7_, extract multi-scale features with strides of 8, 16, 32, and 64, respectively. Then, W-BiFPN adaptively assigns weights to each feature layer through layer-wise feature fusion operations, integrating these multi-scale features into higher-level representations. Finally, the fused multi-resolution feature maps are passed to the connection layer of W-BiFPN, outputting *N*_1_, *N*_2_, *N*_3_, *H*_1_, *H*_2_, and *H*_3_. The expressions for the above process are given in Equations (15)–(20),

(15) $$N_{1}=F_{1}\left(\frac{w_{1}U(B^{PSA}(M_{7}))+w_{2}M_{6}}{w_{1}+w_{2}+\varepsilon }\right),$$

(16) $$N_{2}=F_{2}\left(\frac{w_{3}U(N_{1})+w_{4}M_{5}}{w_{3}+w_{4}+\varepsilon }\right),$$

(17) $$N_{3}=F_{3}\left(\frac{w_{5}U(N_{2})+w_{6}M_{4}}{w_{5}+w_{6}+\varepsilon }\right),$$

(18) $$H_{1}=F_{4}\left(\frac{w_{7}N_{2}+w_{8}D(N_{3})+w_{9}M_{5}}{w_{7}+w_{8}+w_{9}+\varepsilon }\right),$$

(19) $$H_{2}=F_{5}\left(\frac{w_{10}N_{1}+w_{11}D(H_{1})+w_{12}M_{6}}{w_{10}+w_{11}+w_{12}+\varepsilon }\right),$$

(20) $$H_{3}=F_{6}\left(\frac{w_{13}D(H_{2})+w_{14}(B^{PSA}(M_{7}))}{w_{13}+w_{14}+\varepsilon }\right),$$

where $F_{i},\;i=\left\{1,2,3,4,5,6\right\}$ represents the DSConv feature transformation operation, and *B^PSA^* denotes the BPSA module; *U* and *D* indicate upsampling and downsampling operations for resolution matching, respectively; $w_{j},j=\left\{1,2,3,\cdot \cdot \cdot ,14\right\}$ represents learnable parameters that quantify the contribution of feature layers connected to the concat operation; and ε is a regularization factor used to prevent numerical instability caused by a zero denominator. To accelerate model training, this paper introduces weighted normalized feature fusion $\sum_{j}\frac{w_{j}}{\sum_{j}w_{j}+\varepsilon }\cdot L_{j}$, where $L_{j}$ represents the feature layer operation in the equation, as shown in Equation (15). $L_{1}$ and $L_{2}$ correspond to $U(B^{PSA}(M_{7}))$ and $M_{6}$, respectively, and a regularization factor $\varepsilon =1\times 10^{-4}$ is introduced to prevent numerical instability.

W-BiFPN uses a multi-path structure to fuse shallow features and high-level semantic information in infrared images, dynamically assigning weights based on each path’s contribution to detection. The BPSA mechanism enhances deeper feature responses to key regions, improving object detection accuracy in complex scenes while balancing precision and efficiency.

#### 3.2.2. Multi-Scale Object Detection Layers

To address multi-scale object detection in hazy environments, this paper constructs a network architecture with four detection layers at resolutions of 160 × 160, 80 × 80, 40 × 40, and 20 × 20, as illustrated in Figure 8b. The fused feature maps (*N*_3_, *H*_1_, *H*_2_, and *H*_3_) generated by W-BiFPN are mapped to corresponding detection layers, enabling fine-grained multi-scale object localization through hierarchical optimization. Shallow detection layers (e.g., 160 × 160) focus on high-resolution features to capture edge and texture details, improving small object detection accuracy, while deeper layers (e.g., 20 × 20) leverage semantic information to enhance contour modeling for large objects. Each detection layer dynamically fuses shallow details and deep semantic features, adaptively suppressing background noise caused by residual haze and amplifying feature representations of low-contrast objects.

The 160 × 160 detection layer receives the output from *N*_3_, featuring a large receptive field that captures global semantic information and preserves rich contextual features, making it suitable for high-precision detection of small-scale objects.

The 80 × 80 detection layer receives the output from *H*_1_, integrating mid-level semantic features with some shallow features, enhancing detail representation, and primarily handling small- to medium-scale object detection.

The 40 × 40 detection layer receives the output from *H*_2_, leveraging the fusion of deep and mid-level features to significantly enhance semantic representation and object recognition capabilities, primarily for medium-scale object detection.

The 20 × 20 detection layer receives the output from *H*_3_, representing the deepest feature map, which fully extracts global semantic information and maintains strong abstraction capabilities, enabling precise detection of large-scale objects occupying significant image areas.

#### 3.2.3. Efficient Attention Mechanism

**Figure 9:**
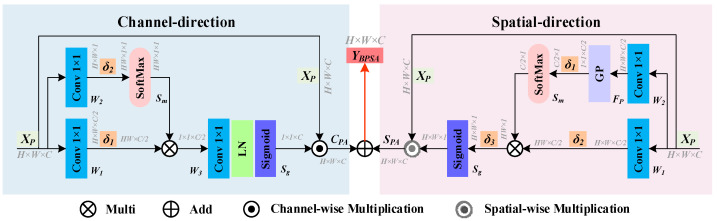
The structure of BPSA, where $X_{p}={\mathbb{R}}^{H\times W\times C}$ represents the input feature map to the BPSA module, and *H*, *W*, and *C* denote the height, width, and channel dimensions of the feature map, respectively.

During the dehazing process, MSFD inevitably leads to some information loss, which may reduce the accuracy of object detection. Therefore, enhancing the robustness and feature extraction capability of the detection model in complex environments is crucial. To address this issue, this paper proposes the bidirectional polarized self-attention (BPSA) mechanism to focus on key regions, effectively distinguishing the object from the complex foggy environment. BPSA establishes long-range dependencies through a joint channel and spatial bidirectional polarized attention mechanism, ensuring high resolution in both the channel and spatial dimensions. The specific structure of BPSA is shown in Figure 9.

The channel-direction polarized attention (CPA) branch realizes the feature optimization in the channel dimension through feature dimensionality reduction, deformation reconstruction, and weighted enhancement. The specific steps are as follows: Suppose the input feature map is $X_{P}\in {\mathbb{R}}^{H\times W\times C}$. First, two-dimensional convolutions with 1 × 1 convolution kernels are used for *W*_1_ and *W*_2_ dimensionality reduction operations, respectively, reducing the number of channels to C/2 and 1 and obtaining the feature maps *G_t_* and *G_r_*. The calculation principles are shown in Equations (21) and (22),

(21) $$G_{t}=W_{1}(X_{P}),$$

(22) $$G_{r}=W_{2}(X_{P}),$$

where $G_{t}\in {\mathbb{R}}^{H\times W\times C/2}$ and $G_{r}\in {\mathbb{R}}^{H\times W\times 1}$; *W*_1_ and *W*_2_ represent two different 1 × 1 convolution transformation functions. Then, tensor transformation operators $\delta_{1}$ and $\delta_{2}$ are used to reshape *G_t_* and *G_r_*, respectively. To enhance the attention range of CPA, the Softmax function is applied to the feature matrix $\delta_{2}(G_{r})\in {\mathbb{R}}^{HW\times 1\times 1}$ obtained from the transformation of $\delta_{2}$, resulting in a correlation weight matrix. This is then weighted with the feature matrix $\delta_{1}(G_{l})\in {\mathbb{R}}^{HW\times C/2}$ obtained from the transformation of $\delta_{1}$ to obtain the feature matrix $Z_{m}$. The computational principle is shown in Equation (23),

(23) $$Z_{m}=S_{m}(\delta_{2}(G_{r}))⊗\delta_{1}(G_{t}),$$

where $Z_{m}\in {\mathbb{R}}^{1\times 1\times \frac{C}{2}}$; $S_{m}$ represents the normalization using the Softmax function. The features $Z_{m}$ are then passed to the feature transformation *W*_3_, which consists of two 1 × 1 convolution layers, LN, and a ReLU. This layer captures the dependencies across channels and increases the dimensionality from C/2 to C. Next, the Sigmoid function normalizes the channel weights $C_{w}\in {\mathbb{R}}^{1\times 1\times C}$ to the range [0, 1]. Finally, a channel-wise multiplication operation is performed between *C_w_* and the input *X_p_* to generate the final channel polarization attention map $C_{PA}\in {\mathbb{R}}^{H\times W\times C}$. The computational principles for the channel weights and the channel polarization attention feature map are shown in Equations (24) and (25), respectively,

(24) $$C_{w}=S_{g}(W_{3}(Z_{m})),$$

(25) $$C_{PA}=C_{w}⊙X_{P},$$

where ⊙ represents the channel-wise multiplication operation.

The spatial-direction polarized attention (SPA) branch models the spatial contextual relationships of the input feature map *X_p_*, dynamically adjusting pixel weights. Pixels with low expression ability are assigned lower weights, while pixels with high expression ability are assigned higher weights. Through pixel-wise weighted fusion, SPA strengthens key spatial location features, suppresses redundant information, builds spatial contextual dependencies in the feature map, and enhances spatial selectivity and feature representation capabilities.

Specifically, *X_p_* is input into two 2D convolution layers with 1 × 1 convolution kernels, generating feature maps $G_{h}\in {\mathbb{R}}^{H\times W\times C/2}$ and $G_{w}\in {\mathbb{R}}^{H\times W\times C/2}$, as expressed in Equations (26) and (27),

(26) $$G_{h}=W_{2}(X_{P}),$$

(27) $$G_{w}=W_{1}(X_{P}).$$

Subsequently, the spatial features in the vertical direction, *G_h_*, are compressed into a spatial feature vector $F_{GP}(G_{h})\in {\mathbb{R}}^{1\times 1\times C/2}$ through global pooling. Then, tensor transformation operators $\delta_{1}$ and $\delta_{2}$ are used to reshape $F_{GP}(G_{h})$ and *G_w_*, producing $\delta_{1}(F_{GP}(G_{h}))\in {\mathbb{R}}^{C/2\times 1}$ and $\delta_{2}(G_{w})\in {\mathbb{R}}^{HW\times C/2}$, respectively. Since spatial features need to be compressed, the Softmax function is applied to enhance the features of $\delta_{1}(F_{GP}(G_{h}))$. Matrix multiplication is then performed on the results to obtain the feature matrix *T_m_*. The computational principle is shown in Equation (28),

(28) $$T_{m}=\delta_{2}(G_{w})⊗S_{m}(\delta_{1}(F_{GP}(G_{h}))),$$

where $T_{m}\in {\mathbb{R}}^{HW\times 1}$; *F_GP_* represents global pooling. Then, after normalizing the tensor transformation operator $\delta_{3}$, the spatial attention weights $S_{w}\in {\mathbb{R}}^{H\times W\times 1}$ and the full-space polarization attention map $S_{PA}\in {\mathbb{R}}^{H\times W\times C}$ are generated. The computational principles are shown in Equations (29) and (30), respectively,

(29) $$S_{w}=S_{g}(\delta_{3}(T_{m})),$$

(30) $$S_{PA}=S_{w}⊙X_{P},$$

where ⊙ represents the spatial multiplication operator.

By combining the bidirectional polarization attention operations in both the channel and spatial directions, the final output $Y_{BPSA}\in {\mathbb{R}}^{H\times W\times C}$ of BPSA is shown in Equation (31),

(31) $$Y_{BPSA}=C_{PA}⊕S_{PA},$$

where ⊕ represents the element-wise addition operation.

The BPSA mechanism improves feature selection by jointly modeling channel and spatial directions, enhancing key regions while suppressing irrelevant background. In the channel dimension, it strengthens inter-channel dependencies through dimensionality reduction, correlation modeling, and per-channel weighting. In the spatial dimension, it emphasizes key pixels and models contextual relationships via spatial context modeling and per-space weighting. By integrating both dimensions, BPSA enhances object detection and localization in complex backgrounds.

#### 3.2.4. Loss Function for Infrared Dehazing Object Detection

In response to the complexity of infrared dehazing object detection tasks, this paper introduces an object detection loss function, DetLoss, which combines scale loss and location loss to address the challenges of detecting objects affected by scale variations and position blurring in foggy environments. Compared to the traditional intersection over union (IoU) loss, DetLoss incorporates dual perception of object scale and position, making it suitable for infrared dehazing object detection tasks.

The scale loss $L_{sca}$ is calculated by integrating the area, IoU, and variance of the object, aiming to measure the degree of match between the predicted object and the ground truth object scale. Its definition is shown in Equation (32),

(32) $$L_{sca}=1-\frac{\min(\left|A_{p}\right|,\left|A_{gt}\right|)+Var(\left|A_{p}\right|,\left|A_{gt}\right|)}{\max(\left|A_{p}\right|,\left|A_{gt}\right|)+Var(\left|A_{p}\right|,\left|A_{gt}\right|)}\cdot \frac{\left|A_{p}\cap A_{gt}\right|}{\left|A_{p}\cup A_{gt}\right|},$$

where $A_{p}$ and $A_{gt}$ represent the pixel sets of the predicted and ground truth objects, respectively; $\left|A_{p}\right|$ and $\left|A_{gt}\right|$ represent the areas of the predicted and ground truth objects; Var(,) is the function for obtaining the scalar variance; and $\frac{\left|A_{p}\cap A_{gt}\right|}{\left|A_{p}\cup A_{gt}\right|}$ represents the IoU between the predicted and ground truth objects. By introducing the area variance function Var(,), the object scale loss effectively measures the scale matching between the predicted and ground truth objects.

The location loss $L_{loc}$ is calculated based on the offset between the predicted center and the ground truth center, modeling the location information in polar coordinates. The center coordinates of the predicted object $A_{p}$ and the ground truth object $A_{gt}$ are defined as $\left(x_{p},y_{p}\right)$ and $\left(x_{gt},y_{gt}\right)$, respectively. After converting the Cartesian coordinates of the centers to polar coordinates, the angular offsets between the predicted and ground truth centers are $\theta_{p}$ and $\theta_{gt}$, while the distance offsets are $d_{p}$ and $d_{gt}$. Their definitions are shown in Equations (33) and (34),

(33) $$\theta_{p}=\arctan(\frac{x_{p}}{y_{p}}),\;d_{p}=\sqrt{x_{p}^{2}+y_{p}^{2}},$$

(34) $$\theta_{gt}=\arctan(\frac{x_{gt}}{y_{gt}}),\;d_{gt}=\sqrt{x_{gt}^{2}+y_{gt}^{2}}.$$

The object location loss $L_{loc}$ is obtained, and its calculation is shown in Equation (35).

(35) $$L_{loc}=\left(1-\frac{\min(d_{p},d_{gt})}{\max(d_{p},d_{gt})}\right)+\frac{4}{\pi^{2}}{\left(\theta_{p}-\theta_{gt}\right)}^{2}.$$

The loss function $L_{DetLoss}$ of IDDNet can be obtained as expressed in Equation (36),

(36) $$L_{DetLoss}=L_{sca}+L_{loc}.$$

DetLoss aims to simultaneously optimize scale consistency and location information matching accuracy in the object detection task, thereby effectively improving infrared object detection performance in foggy environments.

### 3.3. Two-Stage Training Strategy

Most existing infrared dehazing detection networks simply combine the dehazing and detection modules in a cascaded manner, which often fails to fully exploit their synergistic potential [50]. To address this limitation, we propose a two-stage training strategy to optimize model performance. In the first stage, the dehazing module and detection module are trained independently, ensuring that each module achieves optimal weights without mutual interference. In the second stage, the two modules are jointly trained, with the detection loss function globally optimizing the network parameters to enhance the dehazing module’s output features for better detection compatibility. This strategy, through phased optimization and joint training, effectively improves the model’s ability to focus on critical object information in the environment, thereby enhancing the overall performance of dehazing and detection.

**Figure 10:**
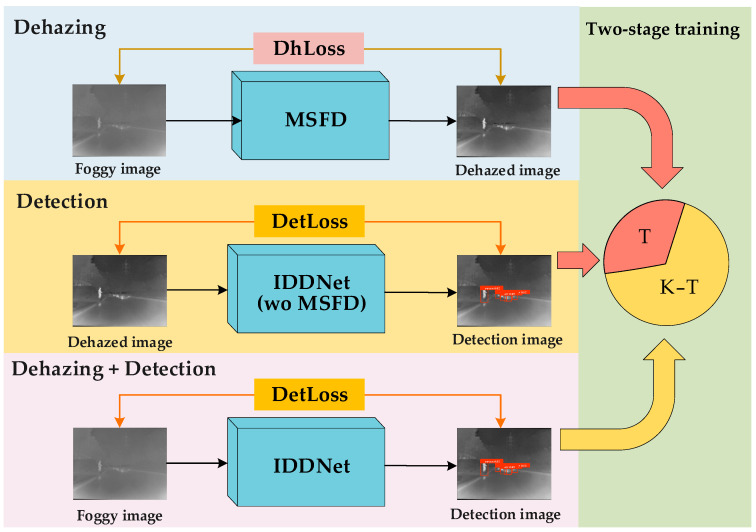
The diagram of the two-stage training strategy. The training strategy can be dynamically adjusted by controlling the value of *T*.

Specifically, assuming the total number of epochs is *E*, the training process is divided into two phases: independent training and joint training. *T* epochs are allocated for independent training, while the remaining epochs are used for joint training. During the independent training phase, the dehazing module and the detection module are trained separately. In the joint training phase, the dehazing and detection modules are integrated and trained together. The flowchart of the two-stage training strategy is shown in Figure 10. Experiments on the value of parameter *T* are provided in Appendix A.

Independent training allows IDDNet’s dehazing and detection phases to train without interference, preserving its detection performance in fog-free scenarios. Distributed training across multiple devices can further reduce training time. Joint training enhances model integration, offering better detection performance and improved computational efficiency compared to independent training. The two-stage strategy also provides flexibility, allowing the adjustment of *T* to meet different task requirements and environments.

## 4. Results and Discussion

### 4.1. Experiment Introduction

#### 4.1.1. Dataset

Currently, there is limited research on infrared dehazing object detection, and the available data samples in this field are scarce. To validate the effectiveness of the proposed IDDNet network, we used the public datasets TVIH [51], IRSTD [52], and the self-constructed infrared foggy dataset TVIN-F [53] for training and evaluation. The TVIH dataset covers multiple scenes with high fog density, but most images do not contain specific infrared objects, so it is primarily used to assess the dehazing performance of the algorithm. The IRSTD dataset contains numerous foggy scenes, while the TVIN-F dataset consists entirely of foggy scenes, with each image containing infrared objects. These datasets were mainly used to evaluate the detection performance and generalization capability of IDDNet.

**Figure 11:**
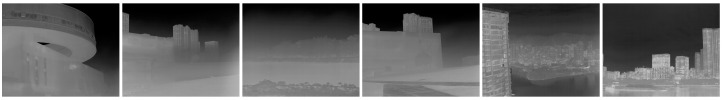
Some representative images from the TVIH dataset.

The TVIH dataset includes 81 high-resolution infrared images depicting varying fog densities across complex backgrounds like roads, water, forests, mountains, and buildings. It contains a few objects such as pedestrians, vehicles, and boats. In this study, 72 images were used for training and 9 for testing, representing light, medium, and heavy fog scenarios (Figure 11).

**Figure 12:**
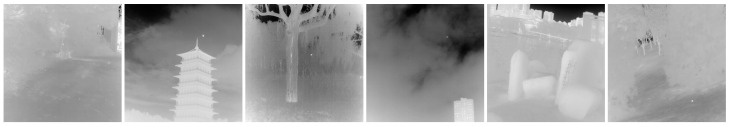
Some representative images from the IRSTD dataset.

The IRSTD dataset consists of 1001 real-world infrared images, with diverse fog densities (clear, light, and heavy fog) and complex backgrounds like forests, buildings, and water surfaces. It also includes small infrared objects from drones at varying altitudes. This dataset was used to evaluate the algorithm’s performance, with 890 images for training and 111 for testing (Figure 12).

**Figure 13:**
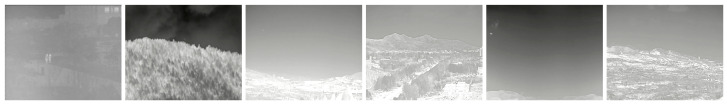
Some representative images from the TVIN-F dataset.

The TVIN-F dataset, containing 1018 infrared foggy images, focuses on complex backgrounds such as mountain ranges, forests, and buildings and features flying infrared small objects. This dataset was used to assess the generalization performance of the algorithm, with 900 images for training and 118 for testing (Figure 13).

#### 4.1.2. Experimental Environment and Training Strategies

The hardware setup and environmental parameters utilized during the experimental training phase are presented in Table 1, and some of the key parameter settings during model training are shown in Table 2.

To enrich the diversity of the data samples, all images in the training set were randomly cropped to a size of 128 × 128 before being input into the model, and random rotation and flipping augmentations were applied.

#### 4.1.3. Evaluation Indicators

To evaluate IDDNet’s overall performance, comprehensive and reliable indicators were selected for both MSFD and IDDNet. For dehazing performance, three indicators were used: information entropy (IE), peak signal-to-noise ratio (PSNR), and structural similarity index (SSIM). For object detection performance, four metrics were used: precision (P), recall (R), AP_50_, and AP_50:95_. Additionally, frames per second (FPS), model size, and number of parameters were used to assess the model’s overall performance.

IE is used to describe the average amount of information retained in the infrared dehazed image. Its mathematical expression is shown in Equation (37),

(37) $$IE=-\sum_{i=0}^{N}P(x_{i})\log_{2}P(x_{i}),$$

where $P(x_{i})$ represents the probability of pixels with a grayscale value equal to *i* and *N* represents the maximum grayscale value of the image.

PSNR is used to evaluate image quality by measuring the difference between the restored image and the original, reflecting the fidelity of the restored image. Its mathematical expression is shown in Equation (38),

(38) $$PSNR=10\times \log_{10}\left(\frac{MAX_{J(x)}^{2}}{MSE}\right),$$

where *MSE* represents the mean squared error and $MAX_{J(x)}^{2}$ represents the square of the maximum pixel value of J(x).

SSIM is used to measure the structural similarity between two images and is commonly used to assess image quality. It considers the brightness, contrast, and structural information of the images to evaluate the quality of the restored image. Its mathematical expression is shown in Equation (39),

(39) $$SSIM(x,y)=\frac{\left(2\mu_{x}\mu_{y}+C_{1})(2\sigma_{xy}+C_{2}\right)}{\left(\mu_{x}^{2}+\mu_{y}^{2}+C_{1})(\sigma_{x}^{2}+\sigma_{y}^{2}+C_{2}\right)},$$

where *x* represents the dehazed image; *y* represents the clear image; *μ* and *σ* represent the mean and variance, respectively; *σ_xy_* represents the covariance; and *C*_1_ and *C*_2_ are constants used to stabilize the formula calculation.

Precision represents the proportion of true positive samples among all the detection samples predicted as positive. This indicator is an important indicator of the accuracy of the positive predictions. Its mathematical expression is shown in Equation (40),

(40) $$Precision=\frac{TP}{TP+FP}.$$

Recall represents the proportion of true positives correctly predicted out of all actual positives. This indicator is used to assess the ability to recall positives. Its mathematical expression is shown in Equation (41),

(41) $$Recall=\frac{TP}{TP+FN},$$

where true positive (*TP*) represents true positives, false positive (*FP*) represents false positives, and false negative (*FN*) represents false negatives.

Average precision (*AP*) provides a comprehensive evaluation of the overall performance of the algorithm, where a higher value indicates better detection effectiveness.

(42) $$AP=\int_{0}^{1}Precision(Recall)d(Recall)$$

AP_50_ represents model performance at a lower IoU threshold, while AP_50:95_ provides a more comprehensive assessment across a range of IoU thresholds from low to high. In infrared object detection scenarios, AP_50_ and AP_50:95_ offer detailed and comprehensive performance evaluations. FPS measures the detection speed of the algorithm, indicating the number of images processed per second. Model size reflects the model’s complexity and storage requirements, primarily determined by the number of parameters and the memory space needed for storage.

### 4.2. Experiment Results

#### 4.2.1. Comparative Experiments of MSFD

To verify the effectiveness and practicality of the proposed MSFD, this section conducted comparative experiments with several representative algorithms in the field of dehazing, including DCP [6], ADE [54], AMEF [55], DehazeNet [10], AMEIF [17], RIDCP [56], C2PNet [57], TIIN [23], and GDN+ [25] models.

DCP is a classic dehazing method based on dark pixel characteristics, widely adopted due to its effectiveness [58,59,60]. ADE uses the CycleGAN framework with U-Net, integrating deep feature extraction, fusion modules, and multi-head attention. AMEF incorporates homomorphic filtering in Gaussian and Laplacian pyramids for rich detail restoration. DehazeNet, a CNN-based model, improves haze-free image quality with bilateral rectified linear units. AMEIF extracts information of fog-free areas by simulating image features under different exposure conditions. RIDCP reduces the domain gap between synthetic and real hazy images using high-quality priors from VQGAN. C2PNet introduces curricular contrastive regularization to enhance lower-bound constraints, using hazy images and restorations from existing methods. TIIN leverages temporal information from neighboring images for enhanced dehazing reliability. GDN+ transfers and fuses information across multiple scales through dense connections. DCP, ADE, and DehazeNet are foundational dehazing algorithms, while AMEIF, AMEF, RIDCP, C2PNet, TIIN, and GDN+ represent innovative advancements. Comparing these methods validates our approach’s effectiveness.

To quantify the performance of various dehazing models, Table 3 presented the validation results of our algorithm and the comparison algorithms on the TVIH test set, measured by the average values of three objective evaluation indicators: PSNR, SSIM, and IE. Bold values represent the best indicator values, and underlined values represent the second-best indicator values. Based on the data presented and compared in Table 3, our algorithm improved PSNR by at least 3.543 dB compared to the best comparative algorithm, GDN+; improved SSIM by at least 0.042 compared to RIDCP; and improved IE by at least 0.376 compared to C2PNet. These results demonstrated that our algorithm outperforms the comparison algorithms in all indicators, showing excellent stability and superiority in the dehazing task.

**Figure 14:**
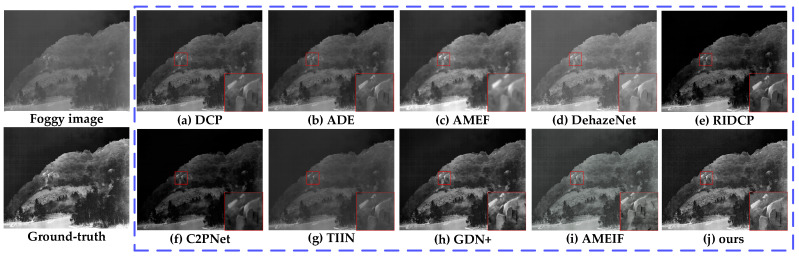
Dehazing results for light fog conditions in Sample 3 of the TVIH test set.

**Figure 15:**
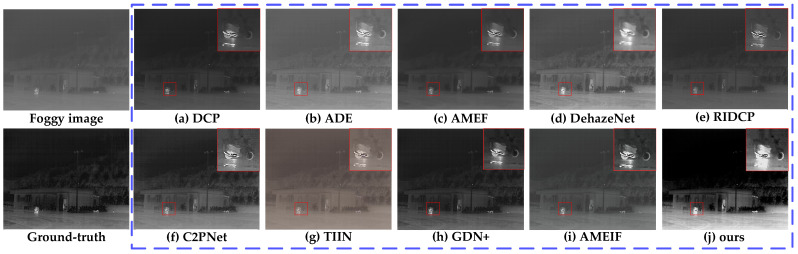
Dehazing results for moderate fog conditions in Sample 6 of the TVIH test set.

**Figure 16:**
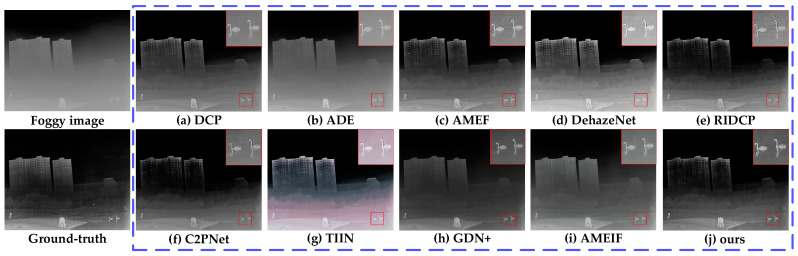
Dehazing results for dense fog conditions in Sample 8 of the TVIH test set.

Figure 14, Figure 15 and Figure 16 showed the dehazing results of different models on infrared images under light, moderate, and dense fog conditions, respectively. From the dehazing results, both the compared models and the proposed model demonstrated certain dehazing capabilities, but there were significant differences in detail restoration, edge sharpness, and color fidelity. The red box in the Figure 14, Figure 15 and Figure 16 indicates the enlarged detail information.

DCP performed well in restoring texture details in light fog conditions (Figure 14a), effectively recovering the structure of rocks. However, its handling of deep fog was insufficient, resulting in noticeable residual fog in moderate (Figure 15a) and dense fog conditions (Figure 16a), leading to overall image darkening and reduced clarity.

ADE could restore the details of rocks and motorcycles quite well in light fog (Figure 14b) and moderate fog (Figure 15b) environments. However, it performed poorly in a dense fog environment (Figure 16b), with a significant amount of fog remaining in some areas, resulting in insufficient permeability and a lack of layering in the image.

AMEF exhibited stability across different fog densities, effectively restoring rock contours in Figure 14c. However, in moderate (Figure 15c) and dense fog (Figure 16c), it suffered from color darkening and detail blurring, particularly in distant regions where its deep fog removal ability was limited.

DehazeNet could remove large areas of fog while maintaining image brightness and contrast, but its overall dehazing performance was average. In light fog (Figure 14d), rock edges remained blurry, while in moderate fog (Figure 15d), residual fog was noticeable on distant trees. In dense fog (Figure 16d), buildings still appeared indistinct.

RIDCP performed consistently across different fog densities with balanced brightness. It effectively restored rock edge details in Figure 14e and recovered textures of houses and ground in Figure 15e. However, in dense fog (Figure 16e), distant areas remained somewhat blurry.

C2PNet delivered balanced performance with good contrast and realistic color restoration. It effectively recovered rock textures and motorcycle contours in Figure 14f and Figure 15f. In Figure 16f, the swan’s silhouette is well-preserved, but background noise was noticeable, and some details remained unrecovered.

TIIN suffered from significant color distortion. In moderate fog (Figure 15g), the image appeared overly yellow and too bright, affecting dehazing realism. In dense fog (Figure 16g), while foreground details were clear, severe color distortion reduced overall visual quality.

GDN+ performed well under light and moderate fog, but when facing heavy fog (Figure 16h), the dehazed image exhibited noticeable shadows, and the removal of large patches of fog was less effective. Additionally, the image texture was not effectively restored.

AMEIF performs better than traditional methods but still lags behind deep learning-based approaches. Fog remains in the images (Figure 15i and Figure 16i), accompanied by some color distortion.

The proposed model achieved the best dehazing performance, demonstrating stability across light, moderate, and dense fog conditions. It significantly enhanced image clarity, as shown in Figure 14j, where rock textures and edge details were well-preserved. In Figure 15j, motorcycle details and building contours were clearly presented, while in Figure 16j, both distant buildings and the swan’s texture were effectively restored.

#### 4.2.2. Ablation Experiments of MSFD

To validate the effectiveness of GCCA, ESA, and the dehazing loss function DhLoss composed of *L_p_*, *L_c_*, and *L_g_* in MSFD, an ablation study was conducted using the TVIH dataset for training and validation. A total of 17 experiments were set up with identical training parameters and configurations. To quantitatively analyze the impact of these modules on dehazing performance, PSNR, SSIM, IE, and FPS were used as evaluation indicators to comprehensively assess the MSFD module. The experimental results are shown in Table 4, where “√” indicates the inclusion of the corresponding module or loss function.

Effectiveness of the GCCA Block: A comparison between Groups 1 and 2 or Groups 14 and 15 shows that the GCCA module outperforms the CCA module in IE (+0.062), with slight improvements in other metrics, confirming the effectiveness of integrating GSConv into the CCA module. The increase in IE indicates better preservation of texture details while reducing noise interference. Although GSConv is a lightweight convolution, since MSFD is built on DSConv, GSConv overcomes DSConv’s limitations in channel information processing by integrating dense convolution operations, making feature extraction and fusion more efficient, thereby improving evaluation metrics. Additionally, GSConv uses shuffle operations to effectively merge information from standard convolutions into the output of depthwise separable convolutions, enhancing the model’s feature capture capability. Moreover, the introduction of the GCCA module not only improves dehazing performance but also increases inference speed (+3.75 FPS).

Experimental comparisons between Groups 12 and 16 demonstrate that the GCCA module plays a key role in infrared image dehazing and detail preservation. The improvements in PSNR (+1.111) and SSIM (+0.05) after adding the GCCA module indicate that it effectively reduces haze interference in image structures and enhances overall detail, leading to better brightness consistency and clearer contours. The increase in IE (+0.18) suggests that the dehazed image contains more valuable information. By improving the feature extraction capability of contrast-aware channels, the GCCA module enhances the model’s adaptability to hazy conditions, effectively suppressing haze interference and accurately restoring image details.

Effectiveness of DhLoss: By removing different components of the dehazing loss function, we evaluated the impact of perceptual loss (*L_p_*), contrast loss (*L_c_*), and smoothness loss (*L_g_*) on model performance and their respective roles. The results indicate that each loss function plays a crucial role in different performance aspects, forming a strong synergistic effect. Experiments from Groups 5, 6, 7, and 9 showed that *L_p_* and *L_c_* significantly improved PSNR (+2.433, +2.247). *L_p_* utilizes high-level features extracted by VGG16, while *L_c_* minimizes local contrast variance. Together, they reduce global brightness shifts caused by haze, making the dehazed image closer to the true radiance distribution and reducing pixel errors. Experiments from Groups 5, 6, 8, and 11 demonstrated that *L_p_* and *L_g_* improved SSIM (+0.02, +0.013), indicating enhanced texture consistency in the dehazed images. The comparison between Groups 5 and 8 revealed that *L_g_* had a particularly noticeable effect on increasing the IE indicator (+0.359). *L_g_* suppresses high-frequency random fluctuations caused by noise while enhancing effective entropy, effectively separating noise from useful details, highlighting its unique advantage in preserving image information integrity.

Specifically, *L_p_* constrains the overall feature distribution of the dehazed image to ensure global structural accuracy. *L_c_* focuses on capturing edge information, improving the clarity of contours in the dehazed image. *L_g_* optimizes gradient distribution to enhance information entropy, helping the dehazed image retain more details. The synergistic effect of these loss functions significantly enhances the dehazing performance of the model, validating the rationality and effectiveness of the loss function design.

Effectiveness of the ESA Block: Experimental results from Groups 12 and 15 show that ESA improves PSNR by 1.452 and SSIM by 0.078. By enhancing the spatial feature representation of key regions, the ESA module more precisely focuses on areas relevant to the object, improving overall image detail extraction. Additionally, ESA integrates stride convolution and large-window pooling operations to expand the receptive field, effectively capturing more contextual information with a slight increase in inference time (FPS decreased by 6.51).

#### 4.2.3. Comparative Experiments of IDDNet

**Figure 17:**
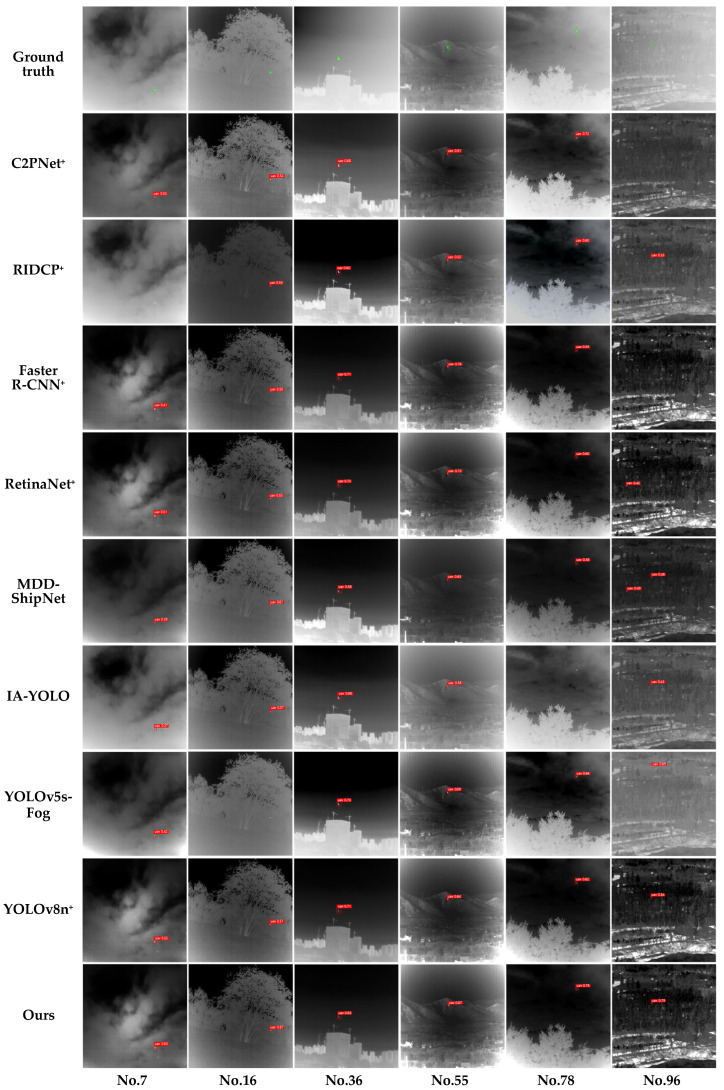
Visualization results of comparison models and our model on the IRSTD dataset.

To comprehensively evaluate the superiority and real-time performance of IDDNet in object detection tasks under hazy scenarios, this section conducted comparative experiments with eight representative algorithms in the field of object detection, including C2PNet^+^ [57] (C2PNet + RTDETR), RIDCP^+^ [56] (C2PNet + RTDETR), Faster R-CNN^+^ (Faster R-CNN + MSFD) [40], RetinaNet^+^ (RetinaNet + MSFD) [39], MDD-ShipNet [28], IA-YOLO [29], YOLOv5s-Fog [30], and YOLOv8n^+^ (YOLOv8n + MSFD). The experimental results, shown in Table 5, present the performance of these algorithms in terms of detection accuracy and real-time capability. Figure 17 shows the object detection results for various models under different haze conditions (light, moderate, and dense fog).

Our model and comparison models were evaluated on the same experimental platform. IDDNet used the two-stage training strategy, while others used the original methods with consistent experimental parameters. We integrated RTDETR as the detection module with C2PNet and RIDCP to form C2PNet^+^ and RIDCP^+^, respectively. The results showed moderate performance for C2PNet^+^ and RIDCP^+^, with large model sizes, especially RIDCP^+^ at 155.33 MB. Although C2PNet and RIDCP showed strong dehazing capabilities, they did not support object detection. Additionally, the dehazing module MSFD was integrated into Faster R-CNN and RetinaNet, forming Faster R-CNN^+^ and RetinaNet^+^. As a classic two-stage detection model, Faster R-CNN+ outperformed RIDCP+ in detection performance, but its need to generate region proposals limited its detection speed to only 20 FPS. RetinaNet+ utilized Focal Loss to dynamically adjust sample weights, improving detection performance over Faster R-CNN+ while achieving better real-time performance. However, its model size remained large at 78.82 MB. MDD-ShipNet achieved good precision (0.829) and recall (0.638) but failed to meet real-time requirements with a frame rate of 60.26 FPS. YOLOv5s-Fog showed good overall performance but had poor stability with AP_50_ and AP_50:95_. IA-YOLO had a larger model and lower performance compared to YOLOv5s-Fog. YOLOv8n, though small, lacked dehazing functionality. When combined with MSFD, YOLOv8n formed YOLOv8n+, which had the smallest size but moderate detection accuracy and weaker stability. In contrast, IDDNet excelled in precision (0.894), recall (0.824), AP_50_ (0.839), and AP_50:95_ (0.551), offering superior detection performance and stability, with a stable processing speed of 74.87 FPS and optimal model size, meeting real-time infrared object detection requirements.

Figure 17 shows the visualization results. The first row shows the ground truth with green bounding boxes; rows two to nine show the detection results of the comparison algorithms, with red bounding boxes indicating detected objects, such as “uav 0.43” indicating a drone with a confidence of 0.43. The 10th row shows IDDNet, which performed the best overall. In contrast, C2PNet^+^ and RIDCP^+^ exhibited missed detections. C2PNet^+^ failed to detect the object in the light fog scene with a complex background (No. 96), while RIDCP^+^ failed to detect the object in the complex foggy environment of No. 7. RetinaNet^+^ provided higher confidence scores than Faster R-CNN^+^, but both models showed missed and false detections in the complex background of No. 96. MDD-ShipNet showed good dehazing performance with no missed detections, but a false detection occurred in the complex background of No. 96. IA-YOLO and YOLOv5s-Fog performed well in scenes with lower fog intensity but still had shortcomings. IA-YOLO failed to detect the object in No. 78 due to incomplete dehazing, while YOLOv5s-Fog missed detections in the overlapping background of No. 16 and falsely detected in the complex background of No. 96. YOLOv8n^+^ did not exhibit significant missed or false detections, but its confidence scores were relatively low, and its performance in more complex scenes like No. 55 and No. 96 was weaker. In comparison, IDDNet performed excellently across various haze intensity scenarios, benefiting from the excellent dehazing performance of the multi-scale fusion dehazing network module, allowing for clear object presentation in hazy scenes. The detection results showed that our algorithm did not have missed or false detections, providing high detection performance and confidence, with the best overall performance and stable, reliable dehazing effects.

#### 4.2.4. Ablation Experiments of IDDNet

To validate the effectiveness of the bidirectional polarized self-attention (BPSA), weighted bidirectional feature pyramid network (W-BiFPN), and the object scale loss *L_sca_* and object position loss *L_loc_* components of DetLoss, an ablation experiment was conducted using the IRSTD dataset for training and validation. A total of 7 experiments were set up, with all training parameters and configurations kept consistent across the groups. To quantitatively analyze the impact of these modules on object detection performance, precision, recall, AP_50_, AP_50:95_, FPS, model size, and number of parameters were used as evaluation indicators. The experimental results are shown in Table 6, where “√” indicates the inclusion of the corresponding module or loss function.

Effectiveness of W-BiFPN: The comparison between Groups 4 and 6 demonstrates that this module enhances contextual information transfer between features through efficient multi-layer feature fusion while improving the model’s adaptability to objects of different scales. The results show that after incorporating W-BiFPN, the IDDNet algorithm experienced a slight precision drop of 0.01, but recall increased significantly by 0.034, AP_50_ improved by 0.024, AP_50:95_ increased by 0.006, and FPS increased by 0.99. In terms of model complexity, the model size increased by 0.16 MB, and the number of parameters grew by 0.05 M. Overall, W-BiFPN enhances the model’s ability to capture fine details through a weighted feature fusion strategy, making multi-scale object features more precise and distinguishable. While the model size slightly increased, detection performance was comprehensively improved.

Effectiveness of BPSA: The comparison between Groups 5 and 6 shows that the addition of the BPSA module increased AP_50_ by 0.021 and significantly improved AP_50:95_ by 0.015, indicating that this module enhances the model’s fine-grained prediction capability for key regions in complex scenes. Additionally, the BPSA module improved recall by 0.028, enhancing the model’s ability to capture critical objects and reducing the risk of missed detections, although FPS slightly decreased. In terms of model complexity, the model size increased by 1.28 MB, and the number of parameters grew by 0.17 M. Overall, the BPSA module improves the extraction of key information and global features, enhancing detection performance. Although it increases algorithm complexity, the improvement in detection accuracy outweighs the computational cost, making the model more stable and effective in complex scenarios.

Effectiveness of DetLoss: In the comparative experiments of Groups 1, 2, 3, and 7, different components of the detection loss function were removed to evaluate the impact of *L_sca_* and *L_loc_* on detection performance. The results show that after introducing *L_sca_*, the area constraint and variance penalty helped reduce false positives and missed detections. Additionally, the variance penalty forced the model to balance learning features of different-sized objects, preventing performance bias caused by an imbalanced scale distribution in the training data. This led to improvements in precision (+0.045), recall (+0.041), and AP_50_ (+0.036), with gains in AP_50:95_ and FPS. After introducing *L_loc_*, the angle offset and distance offset constraints improved precision and recall, enhanced localization accuracy, and increased AP_50_ (+0.041). Overall, DetLoss improves the model’s performance by integrating local and global information, enhancing robustness, particularly for objects with unclear boundaries or significant size variations.

Our model achieved a precision of 0.894 and an AP_50_ of 0.839, with a storage size of 10.35 MB and 2.15 M, running at 74.87 FPS.

#### 4.2.5. Generalization Experiment of IDDNet

This section conducted generalization experiments of IDDNet on the IRSTD and TVIN-F datasets. The parameter settings, training conditions, and other experimental environments for the generalization experiments were consistent with those in the previous experiments to ensure the comparability and consistency of the results. The results are shown in Table 7.

Analysis of the experimental results in Table 7 showed that IDDNet achieved high performance on both the IRSTD and TVIN-F datasets, with particularly outstanding results in precision and recall, indicating its significant reliability and accuracy in infrared dehazing object detection tasks. Further analysis revealed that IDDNet demonstrated good adaptability in AP_50_ and AP_50:95_ across different datasets and scenarios, validating its generalization capability in dehazing object detection tasks. It showed strong robustness in diverse object scales, complex hazy scenes, and varying background conditions.

**Figure 18:**
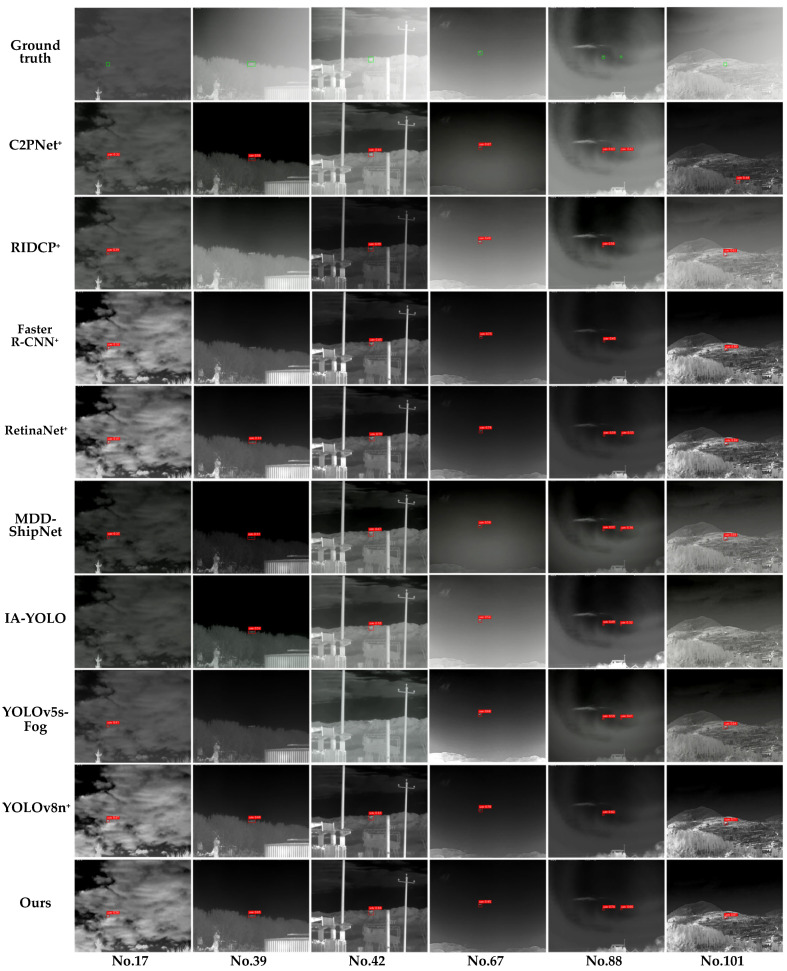
Visualization results of comparison models and our model on the TVIN-F datasets.

The visualization results of the TVIN-F test set are shown in Figure 18. IDDNet performed the best in detecting flying infrared objects under various haze conditions in complex scenes such as cloud cover, forest occlusion, electrical equipment, and mountain ranges. MSFD enabled IDDNet to achieve high confidence in detection results and successfully detect all objects, with no missed or false detections. In comparison, while MDD-ShipNet and RetinaNet^+^ detected all objects, their confidence was relatively low. Additionally, MDD-ShipNet exhibited unstable dehazing performance, with residual fog remaining in scenes No. 88 and No. 101. YOLOv8n^+^ showed good detection results but missed small objects in scene No. 88. RIDCP^+^, Faster R-CNN^+^, and YOLOv5s-Fog failed to accurately detect objects in forest occlusion scenarios such as scene No. 39, and additionally, RIDCP^+^ and Faster R-CNN^+^ missed small objects in scene No. 88, while YOLOv5s-Fog missed objects in scene No. 42 that overlapped with the background. IA-YOLO performed generally and failed to detect objects in the hazy scene No. 17 and the complex background of scene No. 101. C2PNet^+^ had false detection in scene No. 101, indicating its limited ability to handle complex backgrounds.

### 4.3. Discussion

The IDDNet proposed in this paper achieves excellent performance in infrared dehazing object detection, though there are areas for further exploration. IDDNet outperforms advanced dehazing algorithms, with the MSFD module effectively restoring image details, enhancing clarity, and maintaining integrity. It shows superior stability and robustness across varying haze densities. In object detection, IDDNet leads in precision (0.894), recall (0.824), AP_50_ (0.839), and AP_50:95_ (0.551), while maintaining real-time performance at 74.87 FPS and a model size of 10.35 MB. Experiments on different datasets demonstrate its generalization ability, particularly in complex backgrounds and varying fog conditions. However, IDDNet faces limitations, such as scarce infrared dehazing object detection samples, potential degradation in inference speed with increased network complexity, and challenges in detecting small objects under heavy background interference or with similar features. Despite these, IDDNet’s theoretical and practical contributions are significant, providing new methodologies for infrared dehazing object detection and offering reliable support for real-time object detection in foggy environments, especially in drone monitoring and security surveillance.

## 5. Conclusions

This paper presents IDDNet, a multi-scale fusion dehazing object detection network designed for infrared images in foggy conditions. The network integrates the MSFD module with Maxout operations and DSConv to extract global–local features, while the ESA module improves spatial feature distribution. The GCCA block enhances channel attention, and the DhLoss function optimizes dehazing by combining perceptual, contrast, and smoothness losses. Experimental results show MSFD outperforms existing algorithms with improvements of at least 3.543 dB in PSNR, 0.042 in SSIM, and 0.376 in IE, offering stability and robustness in complex foggy environments.

IDDNet addresses infrared object detection in foggy conditions, using the BPSA module to focus on key areas and the W-BiFPN module to improve feature fusion. Four detection layers enhance accuracy and robustness, while the DetLoss function adapts to scale variation and position blurring. A two-stage training strategy integrates dehazing and detection modules, allowing for flexible adaptation to task requirements. IDDNet is highly efficient, with a model size of only 10.35 MB, making it smaller than most existing algorithms. The algorithm excels in detection accuracy, generalization, and real-time performance. Future work will refine the model for more complex foggy environments and explore the use of temporal information to improve robustness in dynamic scenarios while expanding the dataset for further testing.

## Figures and Tables

**Table 1:** Table of hardware platform and training environment parameters.

Parameters	Configuration
CPU	Intel Core i9-13900K
GPU	NVIDIA RTX 4090 GPU
GPU memory size	24 G
Operating systems	Win 10
Deep learning architecture	Pytorch1.9.2 + Cuda11.4

**Table 2:** Some key parameters set during model training.

Parameters	Setup
Epochs	500
*T*	200
Adjustable exponential decay rate *β*_1_	0.9
Adjustable exponential decay rate *β*_2_	0.999
Initial learning rate	0.01
Final learning rate	0.0001
Batch size	4
Input image size	128 × 128
Optimizer	Adam
*L_DhLoss_* (*λ*_1_)	1
*L_DhLoss_* (*λ*_2_)	0.2
*L_DhLoss_* (*λ*_3_)	0.4

**Table 3:** Dehazing results of the classical model and the proposed model (the bold data in the table indicate the best results; “↑” denotes that the higher the value of this indicator, the better the performance of the model; “__” represents the second-best indicator values).

Model	PSNR (↑)	SSIM (↑)	IE (↑)
DCP	17.314	0.683	6.842
ADE	16.949	0.716	7.243
AMEF	20.054	0.831	7.512
DehazeNet	20.768	0.729	7.315
AMEIF	21.343	0.827	7.301
RIDCP	24.017	0.847	7.583
C2PNet	22.336	0.842	7.591
TIIN	20.125	0.806	7.373
GDN+	25.350	0.834	7.396
Ours	**28.893**	**0.889**	**7.967**

**Table 4:** Dehazing results of the ablation experiments of MSFD (bold values represent the best indicator values, and “↑” denotes that the higher the value of this indicator, the better the performance of the model).

No.	CCA	GCCA	*ESA*	*L_p_*	*L_c_*	*L_g_*	PSNR (↑)	SSIM (↑)	IE (↑)	FPS (↑)
1	√	-	-	-	-	-	22.641	0.697	6.801	113.56
2	-	√	-	-	-	-	22.678	0.701	6.823	116.74
3	-	-	√	-	-	-	23.271	0.703	6.821	115.01
4	√	-	√	-	-	-	24.437	0.711	6.876	106.48
5	-	√	√	-	-	-	24.601	0.716	6.885	110.23
6	-	√	√	√	-	-	27.034	0.736	7.184	
7	-	√	√	-	√	-	26.848	0.703	6.993	
8	-	√	√	-	-	√	27.192	0.729	7.244	
9	-	√	√	√	√	-	28.027	0.798	7.332	
10	-	√	√	-	√	√	27.352	0.723	7.291	
11	-	√	√	√	-	√	27.125	0.715	7.081	
**12**	-	√	√	√	√	√	**28.893**	**0.889**	**7.967**	
13	-	-	-	√	√	√	27.333	0.800	7.487	123.09
14	√	-	-	√	√	√	27.423	0.804	7.681	113.56
15	-	√	-	√	√	√	27.441	0.811	7.743	116.74
16	-	-	√	√	√	√	27.782	0.839	7.787	115.01
17	√	-	√	√	√	√	28.507	0.872	7.811	106.48

**Table 5:** Detection results of the classical model and the proposed model (the bold data in the table indicate the best results).

Model	Precision	Recall	AP_50_	AP_50:95_	FPS	Model Size (MB)
C2PNet^+^	0.803	0.561	0.609	0.412	59.62	76.62
RIDCP^+^	0.797	0.556	0.584	0.399	51.83	155.33
Faster R-CNN^+^	0.801	0.545	0.599	0.396	20.21	95.61
RetinaNet^+^	0.814	0.550	0.607	0.401	55.32	78.82
MDD-ShipNet	0.829	0.638	0.666	0.437	60.26	22.25
IA-YOLO	0.813	0.575	0.625	0.429	57.23	137.08
YOLOv5s-Fog	0.824	0.629	0.661	0.434	63.19	43.19
YOLOv8n^+^	0.815	0.567	0.618	0.427	**75.42**	3.02
Ours	**0.894**	**0.824**	**0.839**	**0.551**	74.87	**10.35**

**Table 6:** Detection results of the ablation experiments of IDDNet (bold values represent the best indicator values).

No.	BPSA	W-BiFPN	*L_sca_*	*L_loc_*	Pre.	Rec.	AP_50_	AP_50:95_	FPS	Model Size (MB)	Params/M
1	√	√	-	-	0.817	0.772	0.752	0.436	71.68	10.35	2.15
2	√	√	-	√	0.866	0.818	0.793	0.498	72.39	10.35	2.15
3	√	√	√	-	0.862	0.813	0.788	0.519	72.56	10.35	2.15
4	-	√	√	√	0.858	0.802	0.818	0.523	73.74	9.02	1.97
5	√	-	√	√	0.845	0.796	0.815	0.532	71.83	10.14	2.09
6	-	-	√	√	0.868	0.768	0.794	0.517	72.75	**8.86**	**1.92**
7	√	√	√	√	**0.894**	**0.824**	**0.839**	**0.551**	**74.87**	10.35	2.15

**Table 7:** Detection results of IDDNet on the IRSTD and TVIN-F datasets.

Dataset	Precision	Recall	AP_50_	AP_50:95_
IRSTD	0.894	0.824	0.839	0.551
TVIN-F	0.889	0.817	0.826	0.547

## Data Availability

The data are available from the authors upon reasonable request.

## Appendix A

Researchers should select the value of *T* based on the specific dataset and research model. We recommend conducting parameter exploration experiments for *T* with a step size of 50 and ensuring it does not exceed 50% of the total number of epochs. Some experimental results regarding the parameter *T* are provided in Table A1.

**Table A1:** Detection results with different parameter *T* values on the IRSTD datasets.

*T*	Precision	Recall	AP_50_	AP_50:95_
0	0.891	0.822	0.834	0.549
50	0.887	0.814	0.829	0.542
100	0.893	0.819	0.833	0.547
150	0.897	0.820	0.837	0.544
200	0.894	0.824	0.839	0.551
250	0.707	0.699	0.678	0.445
300	0.654	0.677	0.620	0.427

As *T* increased from 0 to 200, the proportion of independent training epochs gradually increased, and both the dehazing and detection modules performed well without mutual interference. This resulted in improved detection performance, primarily because independent training allowed the dehazing module to better adapt to foggy environments. However, as *T* continued to increase, the detection performance suddenly dropped sharply because the number of joint training epochs became insufficient, leading to a breakdown in cooperation between the dehazing and detection modules. Therefore, we do not recommend setting *T* above 50% of the total number of epochs.
